# Happiness and health behaviors in South Korean adolescents: a cross-sectional study

**DOI:** 10.4178/epih.e2016022

**Published:** 2016-05-31

**Authors:** Su Yeon Kye, Jeong Hyun Kwon, Keeho Park

**Affiliations:** 1 Cancer Information and Education Branch, National Cancer Control Institute, National Cancer Center, Goyang, Korea; 2 Cancer Policy Branch, National Cancer Control Institute, National Cancer Center, Goyang, Korea

**Keywords:** Adolescent, Happiness, Health behavior

## Abstract

**OBJECTIVES:**

We examined the associations between happiness and a wide range of health behaviors in South Korean adolescents.

**METHODS:**

Study data were derived from the ninth Korea Youth Risk Behavior Web-based Survey administered from June to July 2013. In addition to happiness levels, the questionnaire included items on sociodemographics and health-related lifestyle factors (smoking, drinking, eating breakfast, fruit and vegetable consumption, physical activity, sedentary behavior, and hours of sleep).

**RESULTS:**

The multivariate analysis revealed that higher levels of happiness were associated with not smoking or drinking, eating breakfast, eating fruits daily, vegetable consumption, participating in at least 60 minutes of physical activity a day, avoiding sedentary behavior, and hours of sleep. Additionally, sex differences were found in relationships between happiness and eating fruit daily, participation in physical activity, and sedentary behavior.

**CONCLUSIONS:**

These results encourage public health professionals to consider the psychological aspects of adolescent life in working to improve their health behaviors and outcomes.

## INTRODUCTION

Positive psychology has become more prominent in recent years, as empirical evidence confirming the influence of positive psychological variables such as happiness on well-being continues to emerge [1]. Happiness has been broadly used to describe positive subjective experiences, and is often said to comprise two components: the cognitive appraisal of one’s life and affective evaluations (both positive and negative), which are viewed as separate dimensions [2].

Research on happiness has suggested that higher levels of happiness have multiple health benefits, such as a reduced risk of morbidity, disability, and mortality [3]. One possible explanation for the health outcomes of happiness may be that positive well-being may be accompanied by health behaviors that reduce the risk of disease and promote health. Since approximately half of cancer deaths could be prevented by improvements in health behaviors and environmental factors, the investigation of associations between happiness and health behaviors may prove helpful in developing cancer prevention strategies [4].

Although substantial evidence exists that health behaviors are associated with negative mood states, the relationship of healthy lifestyles with happiness is less well established [5,6]. While associations between self-reported happiness and refraining from smoking, lower alcohol consumption, high levels of physical activity, and a healthy diet have been documented in several studies [7], the underlying mechanisms of these effects are still subject to debate, as findings vary among countries, especially between developed and developing nations [8].

Happiness is considered to be particularly important in adolescents, due to its contribution to their future success [9]. However, few studies have investigated adolescent health behaviors in relation to happiness from their perspective. Therefore, after adjusting for known socioeconomic factors, we examined associations between happiness and a wide range of health-related lifestyle behaviors, such as smoking, drinking, eating breakfast, eating fruits and vegetables, physical activity, sedentary behavior, and hours of sleep among South Korean (here after Korean) adolescents. We predicted, on the basis of previous findings, that being a non-smoker or non-drinker, eating breakfast, eating fruits daily, vegetable consumption, participating in physical activity, engaging in less sedentary behavior, and sufficient sleep would be associated with higher levels of happiness.

## MATERIALS AND METHODS

### Participants and procedures

The data were derived from the ninth Korea Youth Risk Behavior Web-based Survey (KYRBS) [10], administered by the Korea Centers for Disease Control and Prevention (KCDC) from June to July 2013. The KYRBS is a self-administered, anonymous, online survey comprising 126 questions across 16 categories, including health behaviors and perceived happiness [10]. In 2013, 75,149 students enrolled in 400 middle schools and 400 high schools were randomly selected to participate in the ninth KYRBS. Each student was randomly assigned a unique identification number, which they used to log into the survey Web page in the computer room of their school. Before they began the questionnaire, an item asked potential respondents to electronically indicate whether they agreed to participate. Those who declined to participate did not proceed further. A total of 72,435 students (36,655 boys, 35,780 girls), whose complete demographic data were obtained through the survey, agreed to participate (response rate, 96.4%). Details of the sampling and scheduling procedures have been described elsewhere [11].

This study was approved by the institutional review board (IRB) of the Korean National Cancer Center (IRB no. NCC2014-0028), and informed written consent was obtained from all study participants.

### Instruments

Happiness was assessed using a single item: ‘In general, how would you describe your happiness?’ The predefined responses were very happy, a little happy, neutral, a little unhappy, and very unhappy. A previous study demonstrated that this single item had good concurrent, convergent, and divergent validity [12]. The happiness measure used in this study most probably relates to overall happiness rather than specific components such as affect or contentment. Single-item happiness measures have been widely used in the literature in several different cultures [13].

Subjective economic status was defined by respondents’ self-reported family incomes. The participants chose one of five descriptors of family income levels: low, low-middle, middle, middle-high, and high income. Current smoking and drinking were determined using the question ‘During the past 30 days, on how many days did you smoke cigarettes/drink alcohol?’ [14]. Those who responded ‘more than one day’ were classified as current smokers and drinkers. Eating patterns were assessed with three questions regarding how frequently each participant had consumed breakfast, fruits, or vegetables over the past seven days. Each item was reclassified as a dichotomized variable reflecting the desired diet variables: breakfast (≥3 times/wk) [15], fruits (≥1 serving/d) [16], vegetables (≥3 serving/d) [16].

Physical activity was evaluated by asking: ‘During the past seven days, on how many days do you engage in any intense activities that cause an increase in breathing or heart rate for a total of at least 60 minutes per day?’ Responses were reclassified into two categories: <3 times/wk and ≥3 times/wk [17]. Sedentary behavior was assessed by asking: ‘On an average school day, for how many hours do you watch TV or videos, or play computer or video games in your leisure time?’ These responses were also categorized into two groups: <2 hr/d and ≥2 hr/d [15]. Sleeping patterns were defined in terms of the number of hours respondents had slept on weekdays and weekends over the previous seven days. Each item was reclassified as a dichotomized variable: <8 hr/night and ≥8 hr/night [18].

The KCDC assessed the reliability of the KYRBS [10], and found that self-reported health risk behaviors among Korean middle and high school students were reliable over time [19].

### Data analysis

Cell percentages (%) are weighted percentages using survey sample weights. The adjusted odds ratios (aORs) and 95% confidence intervals (CIs) in this study were based on weighted analyses. The chi-square test was used to examine differences between boys and girls in sociodemographic characteristics, health behaviors (e.g., smoking, drinking, eating habits, physical activity, and sleeping patterns), and happiness levels. Multivariate logistic regression analysis was performed to determine aORs for happiness levels related to smoking, drinking, eating habits, physical activity, and sleeping patterns by sex, after adjusting for age and subjective economic status. In this study, age was included as a factor for adjustment because adolescence is an age-sensitive period in cognitive, affective, and social development. The data were analyzed using SPSS 15.0 (SPSS Inc., Chicago, IL, USA).

## RESULTS

The sample consisted of 72,435 Korean adolescents between 12 and 18 years of age, 47.7% of whom were girls. Of the respondents, 58.2% perceived themselves as happy (very happy or a little happy). According to sex, the happiness level of boys was significantly higher than that of girls. Approximately 9.7% of adolescents smoked currently and 16.3% were current drinkers. Three-fourths of respondents had breakfast more than three days per a week, and only one-fifth ate fruits daily. The proportion of participants who consumed vegetables more than three days per a week was 16.6%. Approximately 30% of adolescents participated in at least 60 minutes of physical activity more than three days per a week, while 66.3% of them spent more than 2 hours a day watching TV or videos or playing computer or video games in their leisure time. More than 8 hours of sleep were reported by 21.8% of respondents on weekdays and 66.3% on weekends. The health behaviors that showed differences according to sex were current smoking, current drinking, eating fruit daily, vegetable consumption, physical activity, sedentary behavior, and hours of sleep (Table 1).

Table 2 provides aORs for the associations between happiness and current smoking, current drinking, eating breakfast, eating fruit, vegetable consumption, physical activity, sedentary behavior, and hours of sleep by sex. The results indicate that both boys and girls who were happier were less likely to smoke or drink. Girls who were happy were more likely to eat fruit daily and less likely to exhibit sedentary behavior, while boys did not show a significant association between happiness and these behaviors. In contrast, boys who were happy were more likely to participate in physical activity, though no significant relationship was found between happiness and physical activity among girls. Regarding eating breakfast, vegetable consumption, and sleeping patterns, adolescents who perceived themselves as happier were more likely to have healthier lifestyles regardless of sex.

## DISCUSSION

This study was conducted to explore the associations of happiness with health-related lifestyle factors in the Korean adolescent population using a web-based questionnaire survey. The main hypotheses were largely confirmed. Multivariate analysis of the survey data demonstrated that self-reported happiness was significantly associated with the absence of current smoking or drinking, eating breakfast, eating fruits every day, vegetable consumption, participating in at least 60 minutes of physical activity a day, sedentary behavior, and hours of sleep. Additionally, sex differences were found in the relationships between happiness and eating fruit every day, participation in physical activity, and sedentary behavior.

The proportion of adolescents in our study who reported that they were very happy or a little happy with their lives was 58.2%. This proportion was noticeably lower than has been found in Western populations. In a Norwegian survey targeting adolescents participating in a World Health Organization project, the proportion of happy individuals was approximately 90% [9]. In another survey targeting Japanese adults, the proportion of participants reporting being happy was found to be only 48.4% [20]. In contrast, a nationwide survey of US adults reported an overwhelming majority of happy individuals (94.8%) [13]. Further, a previous study found that the probability of being in a depressed mood was greater in collectivistic than in individuaistic cultures [21]. Thus, these findings seem to suggest the presence of lower levels of self-reported happiness among individuals in collectivistic societies such as Japan and Korea relative to individualistic Western societies.

Our results indicated a negative relationship between happiness and smoking or drinking in both boys and girls. These results correspond to previous findings [22]. Adolescents who are not satisfied with their lives smoke and drink more often than those who do not experience these feelings. Our findings also suggest that the associations were stronger in girls than in boys, perhaps because girls are more sensitive to emotional events or problems [23].

Consistent with previous reports, this study suggests the presence of a positive correlation between happiness and a healthy diet [7,24,25]. Kawada et al. [24] previously found that eating breakfast was negatively related to depressive states, supporting our present findings. Our study also found that girls who were very happy ate fruits daily, and both boys and girls who were very happy consumed vegetables more than three times per day. Psychological comfort may lead a person to pay more attention to his or her health and the health value of certain foods. The broaden-and-build theory suggests that positive emotions may trigger an upward spiral that could reinforce positive behavioral patterns such as healthy eating [26]. The sex differences found in associations between happiness and eating fruit daily may be due to the relative lack of concern for health and the lower consumption of fruits among boys relative to girls [27].

With respect to the relationship between happiness and physical activity, we found that boys who were very happy were more likely to participate in at least 60 minutes of physical activity, while a significant association was not found for girls. One possible explanation may be that many girls do not engage in sports, but instead have social interactions outside the sphere of athletics. While several studies have found relationships between reduced participation in sports and emotional difficulties in boys, few have tested for an interaction between sex and sport participation [22]. Contrary to the results for physical activity, the perception of happiness in boys was not related to sedentary behavior, though girls who felt happier were less likely to report sedentary behavior. This may be explained by the fact that boys are less active and tend to enjoy computer games much more than girls, regardless of emotional status.

Our study provided evidence suggesting that happiness may be significantly associated with sufficient sleep, which is consistent with previous findings [28]. Prior research on stress and sleep has focused on the negative consequences of poorer mental health status on sleep disturbance. Zhou et al. [28] demonstrated a longitudinal relationship of stress disorders with sleep problems in adolescents. However, the causal relationship between cognitive and emotional mood and duration of sleep needs to be further clarified, this cross-sectional study suggests whether happiness can totally lead to sufficient sleep in adolescents or not.

The causal pathways linking happiness with health behaviors are still not clear. Bidirectionality is probably involved in the potential relationship between happiness and health behaviors. Several longitudinal studies have suggested that serious and persistent negative emotions were prospective predictors of increased smoking, and, similarly, heavy smoking predicted the subsequent development of depressive symptoms [23]. Previous results of lagged analyses also revealed that consuming fruits and vegetables predicted improvements in positive affect the next day, while, on days when young adults experienced greater positive affect, they reported eating more servings of fruit and vegetables [25]. A similar pattern was found in the area of physical activity. An analysis of the Prospective National Population Health Survey revealed that a change in the status of leisure-time physical activity from active to inactive was associated with increased odds of becoming unhappy two years later [29]. Regarding sleep duration, several longitudinal studies have shown a predictive relationship between disordered sleep and subsequent depression across various populations [30]. Future research should include randomized controlled trials evaluating the influence of happiness on health behaviors in adolescents.

This study had several strengths, including its use of a national representative sample and consideration of a wide range of lifestyle factors. It was also novel in its analysis of adolescents living in Korea, where some of the highest suicide rates in the world have consistently been reported. It had several limitations as well. This study was cross-sectional, so cause–effect relationships could not be confirmed. Our findings were based on self-reports of happiness assessed using a single item; therefore, some of the responses may not have been entirely reliable. With this in mind, a study evaluating the development and evaluation of happiness scales for adolescents could be a next step for future research. Additionally, it is possible that other factors contributed to associations between happiness and health behaviors, such as personality, social environment, or genetic factors. Thus, several additional unmeasured variables may have contributed to the relationships between happiness and health behaviors that we observed. Nevertheless, most importantly, this study demonstrated a meaningful link between two distinct areas of adolescent health research: the literature on happiness levels and the literature on health-related lifestyle factors. Research bridging these two areas is limited, and it is therefore notable that this study identified significant relationships between self-reported happiness and health behaviors.

The present study found that happiness was related to a variety of health behaviors among Korean adolescents, including the absence of current smoking or drinking, eating breakfast, eating fruits daily, vegetable consumption, participating in at least 60 minutes of physical activity a day, sedentary behavior, and hours of sleep. These results encourage public health professionals to take into account the psychological aspects of adolescent life in working to improve their health behaviors and outcomes. Public health strategies promoting health-related lifestyle behaviors among adolescents may easily be improved by encouraging adolescents to appraise their own lives in a positive manner.

## Figures and Tables

**Table 1.** Participant characteristics by sex^1^

Variables^2^	Boys	Girls	Total	X^2^
Total	36,655 (52.3)	35,780 (47.7)	72,435 (100.0)	
Age (yr)				
12-14	16,128 (41.6)	14,992 (41.8)	31,120 (41.7)	0.006
15-18	20,286 (58.4)	20,641 (58.2)	40,927 (58.3)	
Subjective economic status^***^				
High	3,452 (9.7)	1,770 (5.1)	5,222 (7.5)	86.11
Middle-high	9,312 (25.9)	8,213 (23.7)	17,525 (24.9)	
Middle	16,377 (44.7)	18,117 (50.4)	34,494 (47.4)	
Low-middle	5,708 (15.1)	6,098 (16.5)	11,806 (15.8)	
Low	1,806 (4.7)	1,582 (4.2)	3,388 (4.5)	
Current smoking^***^				
Yes	5,349 (14.4)	1,745 (4.6)	7,094 (9.7)	831.20
No	31,306 (85.6)	34,035 (95.4)	65,341 (90.3)	
Current drinking^***^				
Yes	7,139 (19.4)	4,827 (12.8)	11,966 (16.3)	189.83
No	29,516 (80.6)	30,953 (87.2)	60,469 (83.7)	
Eating breakfast (times/wk)				
<3	9,847 (26.7)	9,470 (26.2)	19,317 (26.4)	1.2
≥3	26,808 (73.3)	26,310 (73.8)	53,118 (73.6)	
Eating fruit daily^***^				
Yes	6,650 (18.8)	7,100 (20.8)	13,750 (19.7)	24.09
No	30,005 (81.2)	28,680 (79.2)	58,685 (80.3)	
Vegetable consumption (serving/d)^***^				
<3	30,030 (82.3)	30,218 (84.6)	60,248 (83.4)	50.86
≥3	6,625 (17.7)	5,562 (15.4)	12,187 (16.6)	
Participation in 60 min of physical activity (times/wk)^***^				
<3	21,424 (58.8)	27,715 (77.8)	49,139 (67.9)	1,321.97
≥3	15,231 (41.2)	8,065 (22.2)	23,296 (32.1)	
Sedentary behavior (hr/d)^***^				
<2	12,705 (36.1)	10,512 (30.9)	23,217 (33.7)	106.19
≥2	22,313 (63.9)	23,737 (69.1)	46,050 (66.3)	
Hours of sleep (weekdays, hr/night)^***^				
<8	22,604 (73.5)	26,054 (83.2)	48,658 (78.2)	90.27
≥8	9,012 (26.5)	5,637 (16.8)	14,649 (21.8)	
Hours of sleep (weekends, hr/night)^**^				
<8	10,788 (34.9)	9,895 (32.4)	20,683 (33.7)	8.55
≥8	21,032 (65.1)	21,420 (67.6)	42,452 (66.3)	
Happiness level^***^				
Very unhappy	644 (1.7)	626 (1.7)	1,270 (1.7)	169.96
A little unhappy	3,028 (8.4)	4,067 (11.4)	7,095 (9.8)	
Neutral	10,091 (27.7)	11,852 (33.0)	21,943 (30.2)	
A little happy	13,563 (37.2)	13,154 (37.1)	2,617 (37.1)	
Very happy	9,329 (25.0)	6,081 (16.9)	15,410 (21.1)	

Values are presented as number (%).

1 Weighted percentage using survey sample weights.

2 p-values were determined using the chi-square test.

** p<0.01,

*** p<0.001.

**Table 2.** Associations between happiness and health behaviors^1^

Health behavior/happiness level	Boys	Girls
Current smoking: yes		
Very unhappy	1.00 (reference)	1.00 (reference)
A little unhappy	0.62 (0.51, 0.76)	0.62 (0.47, 0.82)
Neutral	0.57 (0.48, 0.69)	0.36 (0.28, 0.48)
A little happy	0.41 (0.34, 0.49)	0.21 (0.16, 0.27)
Very happy	0.36 (0.30, 0.44)	0.16 (0.11, 0.21)
Current drinking: yes		
Very unhappy	1.00 (reference)	1.00 (reference)
A little unhappy	0.81 (0.67, 0.99)	0.60 (0.49, 0.74)
Neutral	0.70 (0.58, 0.84)	0.41 (0.34, 0.50)
A little happy	0.57 (0.47, 0.69)	0.32 (0.26, 0.39)
Very happy	0.52 (0.43, 0.63)	0.24 (0.19, 0.30)
Eating breakfast: ≥3 times/wk		
Very unhappy	1.00 (reference)	1.00 (reference)
A little unhappy	1.27 (1.05, 1.54)	1.39 (1.15, 1.68)
Neutral	1.29 (1.08, 1.54)	1.59 (1.32, 1.92)
A little happy	1.57 (1.31, 1.87)	2.20 (1.83, 2.63)
Very happy	1.52 (1.27, 1.81)	2.26 (1.87, 2.73)
Eating fruit daily: yes		
Very unhappy	1.00 (reference)	1.00 (reference)
A little unhappy	0.84 (0.68, 1.04)	0.82 (0.65, 1.03)
Neutral	0.73 (0.60, 0.86)	0.85 (0.68, 1.07)
A little happy	0.80 (0.66, 0.99)	0.98 (0.78, 1.23)
Very happy	1.10 (0.90, 1.34)	1.39 (1.11, 1.75)
Vegetable consumption: ≥3 serving/d		
Very unhappy	1.00 (reference)	1.00 (reference)
A little unhappy	0.86 (0.67, 1.09)	0.85 (0.65, 1.10)
Neutral	0.82 (0.65, 1.03)	0.91 (0.71, 1.17)
A little happy	0.94 (0.75, 1.18)	1.05 (0.82, 1.35)
Very happy	1.53 (1.22, 1.92)	1.49 (1.16, 1.93)
Participation in 60 min of physical activity: ≥3 times/wk		
Very unhappy	1.00 (reference)	1.00 (reference)
A little unhappy	0.87 (0.74, 1.03)	0.80 (0.66, 0.98)
Neutral	0.84 (0.72, 0.99)	0.75 (0.62, 0.92)
A little happy	1.05 (0.89, 1.24)	0.83 (0.68, 1.02)
Very happy	1.17 (1.00, 1.37)	1.06 (0.87, 1.30)
Sedentary behavior: ≥2 hr/d		
Very unhappy	1.00 (reference)	1.00 (reference)
A little unhappy	1.24 (1.01, 1.52)	0.90 (0.73, 1.12)
Neutral	1.09 (0.91, 1.31)	0.79 (0.65, 0.97)
A little happy	1.12 (0.93, 1.35)	0.78 (0.63, 0.96)
Very happy	0.97 (0.80, 1.16)	0.72 (0.58, 0.89)
Hours of sleep (weekdays): ≥8 hr/night		
Very unhappy	1.00 (reference)	1.00 (reference)
A little unhappy	1.45 (1.10, 1.90)	1.25 (0.91, 1.72)
Neutral	1.98 (1.53, 2.56)	1.30 (0.95, 1.78)
A little happy	2.29 (1.76, 2.96)	1.66 (1.21, 2.29)
Very happy	3.00 (2.30, 3.91)	2.32 (1.69, 3.19)
Hours of sleep (weekends): ≥8 hr/night		
Very unhappy	1.00 (reference)	1.00 (reference)
A little unhappy	1.27 (1.04, 1.54)	1.15 (0.93, 1.43)
Neutral	1.47 (1.23, 1.77)	1.37 (1.11, 1.69)
A little happy	1.56 (1.30, 1.87)	1.54 (1.26, 1.90)
Very happy	1.63 (1.35, 1.96)	1.72 (1.40, 2.12)

Values are presented as odds ratio (95% confidence interval).

1 Adjusted for age and subjectively reported economic status.
